# Workload of horses on a water treadmill: effect of speed and water height on oxygen consumption and cardiorespiratory parameters

**DOI:** 10.1186/s12917-017-1290-2

**Published:** 2017-11-28

**Authors:** Persephone Greco-Otto, Stephanie Bond, Raymond Sides, Grace P. S. Kwong, Warwick Bayly, Renaud Léguillette

**Affiliations:** 1 Department of Veterinary Clinical and Diagnostic Sciences, Faculty of Veterinary Medicine, University of Calgary, Calgary, AB T2N 4N1 Canada; 2 Department of Veterinary Clinical Sciences and College of Veterinary Medicine, Washington State University, Pullman, WA USA

**Keywords:** Horses, Water treadmill, Conditioning, Rehabilitation, V̇O_2_, Heart rate, Sports physiology

## Abstract

**Background:**

Despite the use of water treadmills (WT) in conditioning horses, the intensity of WT exercise has not been well documented. The workload on a WT is a function of water height and treadmill speed. Therefore, the purpose of this study was to determine the effects of these factors on workload during WT exercise.

Fifteen client-owned Quarter Horses were used in a randomized, controlled study. Three belt speeds and three water heights (mid cannon, carpus and stifle), along with the control condition (dry treadmill, all three speeds), were tested. Measured outcomes were oxygen consumption (V̇O_2_), ventilation (respiratory frequency, tidal volume (V_T_)), heart rate (HR), and blood lactate. An ergospirometry system was used to measure V̇O_2_ and ventilation. Linear mixed effects models were used to examine the effects of presence or absence of water, water height and speed (as fixed effects) on measured outcomes.

**Results:**

Water height and its interaction with speed had a significant effect on V̇O_2_, V_T_ and HR, all peaking at the highest water level and speed (stifle at 1.39 m/s, median V̇O_2_ = 16.70 ml/(kg.min), V_T_ = 6 L, HR = 69 bpm). Respiratory frequency peaked with water at the carpus at 1.39 m/s (median 49 breaths/min). For a given water height, the small increments in speed did not affect the measured outcomes. Post-exercise blood lactate concentration did not change.

**Conclusions:**

Varying water height and speed affects the workload associated with WT exercise. The conditions utilized in this study were associated with low intensity exercise. Water height had a greater impact on exercise intensity than speed.

## Background

Water treadmills (WTs) have historically been used in human athletic training as well as rehabilitation, and are currently gaining popularity in the equine veterinary world. WTs produce low-impact, high resistance work with the ability to minimize concussive forces due to the unique properties of water [1]. Water treadmill manufacturers’ protocols are primarily designed for rehabilitation but in practice, are being used also for conditioning purposes. Despite the popularity of WTs for conditioning of horses, the intensity of WT exercise has not been well documented. In contrast to traditional treadmills in which speed and the slope of the belt determine exercise intensity for an individual horse, the relative workload on a WT is a function of water height and treadmill speed. Weight reduction of horses in water has been estimated to be anywhere from 10 to 30% depending on the water depth [1]. Additionally, the viscosity of water results in high degrees of drag, which reduces the speed by one half to one third for the same energy expenditure [2]. Past research on horses has attempted to describe the intensity of the exercise imposed by the WT by measuring heart rate (HR) [3–6] and/or whole blood lactate concentrations [6, 7], or with muscle biopsies [8, 9] and muscle activation via thermography [10]. However, water temperature, pressure and buoyancy must be taken into account when interpreting data both in horses [4, 6, 7] and humans [11–14]. Additionally, while manufacturers’ protocols are focused on the use of WTs for equine rehabilitation, to our knowledge there are currently no protocols available for conditioning horses on them. Instead, conditioning is reliant on the experience and ability of the operator to gauge fatigue. Two studies from one research group have attempted evaluate the effects of WT conditioning on horses’ HR responses to a specific high-speed treadmill exercise test. They also evaluated the effects of conditioning on the oxidative and glycolytic capacities of the superficial digital flexor and gluteal muscles. After a 4 week training period, there were no changes in the oxidative and glycolytic capacity, or the metabolite/substrate concentrations of both the superficial digital flexor and gluteal muscles [8]. There were also no cardiocirculatory changes following training. The authors concluded that a more strenuous protocol (involving higher speeds and/or longer durations) would be required to improve fitness [8]. The second study conducted by this group used an 8 week training protocol, and found that training resulted in only minor changes in type I muscle fibre sizes [9]. Heart rate and muscle metabolic responses to high-speed treadmill tests did not change [9].

To develop effective conditioning protocols, the effect that water height and treadmill speed have on workload must be better understood. Water height dictates intensity of exercise, since increased water height results in increased resistance to limb movement [11, 15]. However, this is only true up to a certain point – above waist-deep water in humans, metabolic demand begins to decrease, as shown by decreased oxygen consumption (V̇O_2_) [11]. This is because as submersion increases, resistance is partly counteracted by increased buoyancy [11]. While it appears that optimal conditioning occurs when the water is at mid-thigh height for humans [15], an optimal water height has not been determined for horses.

The goal of this study was therefore to determine the effects of water height and speed on exercise intensities (cardiac and respiratory parameters) for horses walking on a WT. We hypothesize that workload during exercise will increase with increasing water height and treadmill speed. The specific objectives were to assess the effect of increasing water height and treadmill speed on V̇O_2_, ventilation, HR and LA accumulation measured during exercise. It is expected that this information will facilitate more objective design of WT-based conditioning protocols for horses.

## Methods

### Horses

Fifteen client-owned Quarter horses (Median age: 14.5 years (Quartiles: 11.5-15.3)) were evaluated during WT exercise in the spring. Horses were all competitive barrel racers and had been out of training during the winter (Median: 3.0 months off (1.8-3.3)). Body weight (Median: 516.9 kg (IQR: 475.5-555.0 kg)) and anatomical heights with the horse standing square on a concrete surface (measured from the ground with an inflexible measuring tape) were recorded. Horses were of similar size and proportions (mid cannon height – 29.2 cm (27.9-30.5 cm); carpus height – 43.2 cm (43.2-45.7 cm); stifle height – 83.8 cm (83.8-88.9 cm); withers height – 152.4 cm (148.0-155.6 cm); or, as a percentage of withers height: mid cannon – 19.2% (18.8-19.8%); carpus – 29.0% (28.3-29.3%); stifle – 56.9% (54.7-57.4%)). All horses were at the WT facility for conditioning and had no history of recent lameness, health issues or poor performance. Horses were examined for lameness and respiratory disease on the dry treadmill, and cardiovascular pathologies were ruled out by ECG analysis by a board certified veterinary internal medicine and sports medicine and rehabilitation specialist. Horses were voluntarily enrolled in the study and owners completed a consent form. This study was approved by the University of Calgary Veterinary Sciences Animal Care Committee.

### Water treadmill conditioning protocol

This was a prospective, randomized, controlled study using repeated measures on all 15 horses. All conditioning and measurements occurred at a private rehabilitation and conditioning facility in Alberta, Canada. All horses were stabled on-site and were acclimated to the treadmill^1^ prior to commencement of the study. Acclimation occurred over three sessions of 20 min. Session 1: Horses were walked at a comfortable pace on the treadmill without any water. Session 2: Horses were walked with water up to the height of the carpus. Session 3: Horses were walked with water up to the stifle. Horses were judged to be acclimated to WT exercise when their movement became regular and they no longer displayed signs of stress. Horses were not sedated at any time during the study.

Three low speeds were chosen for data collection, which allowed horses to walk at a comfortable pace, and ensured consistency across horses. Based on the facility manager’s experience and current practice for WT operations, the three speeds deemed to be appropriate for all horses were 1.11, 1.25, 1.39 m/s. Water height was determined using anatomical landmarks (Mid cannon (at the mid point between the ventral aspect of the accessory carpal bone and the middle height of the lateral sesamoid bone), Carpus (ventral aspect of the accessory carpal bone) or Stifle (proximal point of the patella)) rather than absolute values. The dry treadmill (no water) was used as a control condition. Data was collected daily for three consecutive days on each horse as follows: On each day, the parameters (V̇O_2_, ventilation, HR, lactate) were measured for the control condition (no water) at all three speeds incrementally (1.11, 1.25, 1.39 m/s, in this order); the treadmill was then filled to a specified height (mid cannon, carpus or stifle) and all three speeds were again tested at that height. Speeds were increased incrementally from the slowest speed each experimental day. Post-exercise control measurements at each speed were recorded after water exercise was complete. The experiment was repeated the next day with a different water height. The order of water heights measured was randomized using data management software^2^ and only 1 session per horse was undertaken each day so that all three water heights (and three speed conditions for each water height) were assessed within 3 days for each horse (Table 1). Horses worked on the treadmill for 21 min per session, which included the control (two min per speed, pre- and post-exercise) and exercise with water (three min per speed), but excluded filling and emptying time of the treadmill (5–10 min depending on water height, horses walked continuously). Anatomical locations were identified on the horses while standing and their height from the ground measured and then marked on the WT to ensure consistent filling during WT exercise.

**Table 1:** Randomization of the order for water heights on a water treadmill for 15 horses

Horse	Mid cannon	Carpus	Stifle
**1**	Day 3	Day 1	Day 2
**2**	Day 1	Day 2	Day 3
**3**	Day 2	Day 1	Day 3
**4**	Day 3	Day 1	Day 2
**5**	Day 3	Day 1	Day 2
**6**	Day 2	Day 1	Day 3
**7**	Day 1	Day 2	Day 3
**8**	Day 1	Day 3	Day 2
**9**	Day 2	Day 3	Day 1
**10**	Day 1	Day 2	Day 3
**11**	Day 3	Day 2	Day 1
**12**	Day 2	Day 1	Day 3
**13**	Day 3	Day 1	Day 2
**14**	Day 2	Day 3	Day 1
**15**	Day 3	Day 1	Day 2

One water height test was performed per day, with 24 h to recover between each test. Three water heights were tested (mid cannon, Carpus, Stifle) and the order was set randomly for each horse as described in the table

### Measured outcomes

#### Oxygen consumption and ventilation

**Fig. 1:**
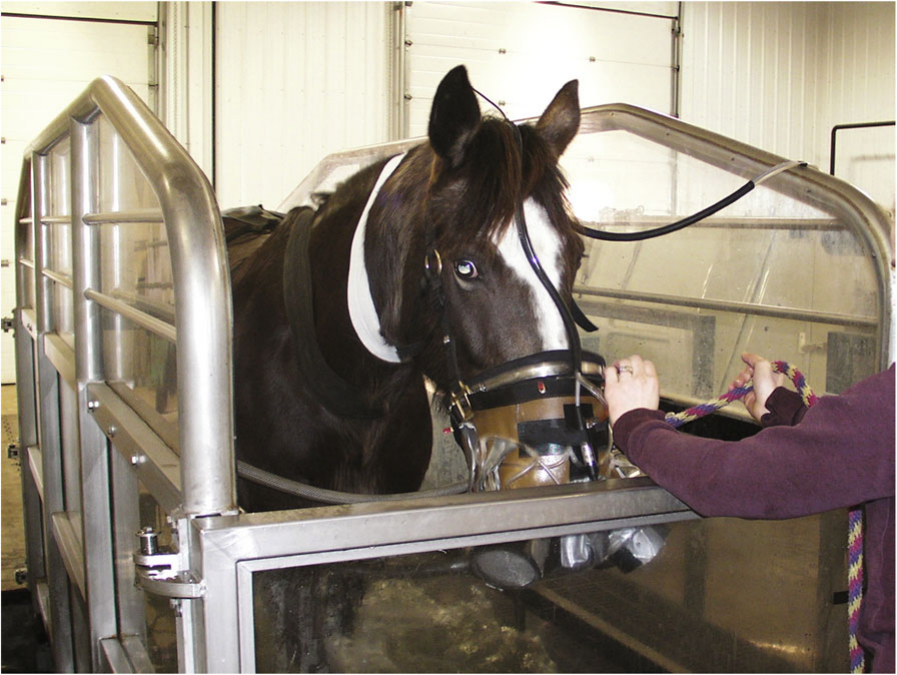
Ergospirometry system using a facemask on a horse exercising in the water treadmill

Oxygen consumption (V̇O_2_) and ventilatory variables (respiratory frequency (RF), tidal volume (V_T_), minute ventilation (V̇_E_,) and the ratio of expiratory duration to inspiratory duration (t_E_/t_I_) were measured continuously using an ergospirometer.^3^ This system reflects a new breath-by-breath approach to the measurement of V̇O_2_ in the field (Fig. 1) [16]. Calibration of the system (flowmeter and gas analyzer) was conducted according to the manufacturer’s instructions before each horse was exercised. The mask was internally padded and adjusted for each horse to minimize the amount of dead space. Results were calculated using customized software provided with the system. Breath-by-breath recordings were analyzed for the final 30 s of each exercise condition after a steady state had been reached. Environmental conditions (ambient temperature, barometric pressure and humidity) were monitored and factored into ventilation calculations. Water temperature data was also collected.

#### Heart rate

Heart rate (HR) was monitored continuously during exercise using a telemetric ECG device and software.^4^ A base/apex configuration was used with three leads on the left side, and one lead on the right side. Mean HR was calculated over the final 60 s of exercise at each speed.

#### Lactate

Jugular venous blood samples (2 ml) were collected in vacutainer tubes containing potassium oxalate at the end of each exercise condition. A handheld analyzer^5^ was used to immediately measure the blood lactate concentration. Accuracy of the analyzer was checked at the beginning of each day by following the manufacturer’s recommendations.

#### Statistical analysis

Linear mixed effects models were used to examine the effects of presence or absence of water, water height and speed (as fixed effects) on V̇O_2_, RF, V_T,_ V̇_E_ and HR (as outcomes), after accounting for the nested data structure from horses (as a random effect). The assumptions of normality and equal variance were assessed for these models. Analyses were performed using R version 3.3.2, and ‘nlme’ package version 3.1 was used for linear mixed effects models analysis. Statistical significance was set at *p* ≤ 0.05 for all tests. All values are reported as median and interquartile range (IQR) to accommodate non-normal data.

## Results

All horses tolerated the WT acclimation and training well and none had to be excluded due to behavioural issues, muscle soreness or lameness.

Environmental conditions were as follows: ambient temperature – 16.2 °C (15.0-16.5); water temperature – 14.0 °C (13.0-15.0); barometric pressure – 757 mmHg (754–764)); humidity – 63% (45–72).

### Linear mixed effects models

The assumptions of normality and equal variance of the tested variables were met for linear mixed effects models.

### Oxygen consumption

**Fig. 2:**
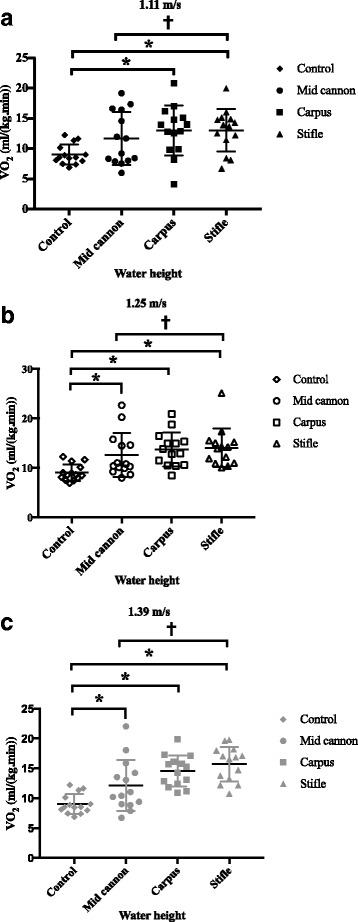
Oxygen consumption (V̇O_2_ in ml/(kg.min)) at varying water heights and belt speeds of 15 horses exercised on a water treadmill. Control is without water. Horizontal bars show median, vertical whiskers show 5-95% percentile. Asterisks (*) denote significant differences. **a** Oxygen consumption with speed set to 1.11 m/s. **b** Oxygen consumption with speed set to 1.25 m/s. **c** Oxygen consumption with speed set to 1.39 m/s

One horse’s oxygen data was unusable due to a malfunction of the mask, therefore the sample size was 14 for all V̇O_2_ and ventilatory data. Results from the linear mixed effects model analysis showed that the presence of water (*p* = 0.006), the water height (*p* = 0.0005), and the interaction for the presence of water and speed (*p* = 0.05) were statistically significant, although the significance of the effect of the interaction between the presence of water and speed was only marginal. Overall, considering interactive effects, V̇O_2_ was greater at a speed of 1.39 m/s compared to 1.11 m/s, regardless of water height. Furthermore, differences in V̇O_2_ between speeds of 1.11 and 1.25 m/s were dependent on the height of the water. Under the control (no water) condition, V̇O_2_ did not differ with changes in treadmill speed (Fig. 2).

All results pertaining to V̇O_2_ and ventilation are summarized in Table 2. V̇O_2_ was greater when horses were exercised in water (all heights) at any speed when compared to control (no water) values, except for the lowest water height and speed combination (mid cannon, 1.11 m/s) (Fig. 2, Table 2). Water height also affected V̇O_2_ – when water was at the height of the stifle, V̇O_2_ was greater than when water was at mid cannon height, regardless of treadmill speed (*p* = 0.005) (Fig. 2). However, there were no differences between V̇O_2_ measurements for water levels at the mid cannon and carpus levels or between carpus and stifle water levels, regardless of speed (Fig. 2, Table 2).

**Table 2:** Ventilatory parameters of Quarter Horses walking on a water treadmill

		Control (no water)	Mid cannon	Carpus	Stifle
V̇O_2_	1.11 m/s	9.57 (6.86-11.88)	10.44 (8.09-15.96) ✝ (*p* = 0.005)	13.44 (10.53-15.05) *(*p* = 0.0002)	13.78 (11.64-14.85) * (*p* < 0.0001)
	1.25 m/s	9.22 (7.01-10.85)	10.57 (9.96-14.55) *✝ (*p* = 0.002, p = 0.005)	13.35 (10.80-15.25) *(*p* < 0.0001)	14.60 (11.18-15.13) *(*p* < 0.0001)
	1.39 m/s	8.93 (6.72-11.26)	10.72 (9.11-14.06) *✝ (*p* < 0.0001, p = 0.005)	14.73 (12.58-16.00) *(*p* < 0.0001)	16.70 (13.42-17.76) *(*p* < 0.0001)
RF	1.11 m/s	37.6 (28.3-46.6)	38.3 (30.9-61.9) ✝ (*p* < 0.0001)	51.9 (39.1-57.0) ✝ (*p* < 0.0001)	33.5 (22.6-43.3)
	1.25 m/s	41.2 (28.4-54.1)	45.0 (37.1-73.6) ✝ (*p* < 0.0001)	51.7 (46.1-60.5) ✝ (*p* < 0.0001)	35.7 (20.8-41.7)
	1.39 m/s	39.7 (28.6-52.5)	47.1 (40.9-76.1) ✝ (*p* < 0.0001)	49.0 (46.1-63.5) ✝ (*p* < 0.0001)	31.5 (21.6-42.9)
V_T_	1.11 m/s	3.8 (3.4-4.3)	4.2 (3.8-4.6) ✝ (*p* < 0.0001)	4.3 (3.8-4.7) ✝ (*p* < 0.0001)	5.1 (4.1-6.1) *(*p* < 0.0001)
	1.25 m/s	3.9 (3.3-4.3)	4.4 (3.7-5.0) *✝ (*p* = 0.02, *p* < 0.0001)	4.6 (4.3-5.1) * ✝ (*p* = 0.007, *p* < 0.0001)	5.5 (4.6-6.1) *(*p* < 0.0001)
	1.39 m/s	3.9 (3.4-4.2)	4.5 (3.9-4.8) ✝* (*p* < 0.0001, *p* < 0.0001)	5.0 (4.5-5.3) * ✝ (*p* < 0.0001, *p* < 0.0001)	6.0 (5.0-7.3) *(*p* < 0.0001)
V_E_	1.11 m/s	157.7 (109.5-198.8)	178.4 (125.5-289.6) ✝ (*p* = 0.004)	218.4 (154.7-265.7) * ✝ (*p* = 0.02, *p* < 0.0001)	169.3 (135.8-238.0)
	1.25 m/s	152.1 (116.5-207.4)	193.9 (150.9-307.3) * ✝ (*p* = 0.001, *p* = 0.004)	242.2 (199.9-265.5)* ✝ (*p* = 0.0002, *p* < 0.0001)	180.4 (132.6-232.7) *(*p* = 0.04)
	1.39 m/s	158.3 (124.0-219.2)	200.0 (184.3-312.1) * ✝(*p* < 0.0001, *p* = 0.004)	261.6 (216.2-290.8) * ✝ (*p* < 0.0001, *p* < 0.0001)	210.0 (159.4-233.0) * (*p* = 0.003)
T_E_/T_I_	1.11 m/s	0.9 (0.8-1.1)	0.8 (0.6-1.0)	0.8 (0.8-0.9)	0.9 (0.8-1.0)
	1.25 m/s	0.9 (0.8-1.0)	0.8 (0.7-0.9)	0.8 (0.7-1.0)	0.8 (0.7-0.9)
	1.39 m/s	0.8 (0.7-0.9)	0.7 (0.7-0.9)	0.8 (0.6-0.9)	0.8 (0.6-0.9)

Ventilatory parameters (V̇O_2_ = oxygen consumption in ml/(kg.min), RF = respiratory frequency in breaths/min, V_T_ = tidal volume in L, V_E_ = minute ventilation in L/min, T_E_/T_I_ = ratio of exhalation over inhalation periods) while working on a water treadmill at three water levels (mid cannon, carpus, stifle) and speeds (1.11, 1.25, 1.39 m/s) compared to control (no water). Control values are averaged over the values collected pre- and post-water exercise each day (3 days). *N* = 14 (one horse’s data was unusable). Median (IQR) shown. Asterixes (*) denote values that are significantly different from control values; crosses (✝) denote values that are significantly different from stifle values

### Ventilation

#### Respiratory frequency

Exercise speed had no effect on RF in the control (no water) condition. Water exercise had no effect of RF when compared to control values (Table 2). However, when the effect of water level on RF at a given speed was considered, values recorded with water at the stifle level were lower than those at mid cannon (*p* < 0.0001) and carpus levels (p < 0.0001) (Table 2). Furthermore, the lowest (median) RF was measured with water at the height of the stifle, and the highest (median) RF was measured with water at the height of the carpus (*p* < 0.0001).

#### Tidal volume

In control conditions (no water) V_T_ was unchanged over the course of the study and was also unaffected by treadmill speed (Table 2). When water was at the level of the stifle, V_T_ was greater than the control condition at all three speeds (*p* < 0.0001). For water at the mid cannon and carpus levels, V_T_ was greater than the control conditions at 1.25 and 1.39 m/s (mid cannon: *p* = 0.02, *p* = 0.0001; carpus: *p* = 0.01, *p* < 0.0001 respectively) (Table 2).

When the effects of water level were considered, the V_T_ with water at the level of the mid cannon and carpus level were less than V_T_ at the level of the stifle (*p* < 0.0001 for both) (Table 2).

#### Minute ventilation

Minute ventilation values are shown in Table 1. They did not change with increases of speed with the control (no water) condition or at any given water level. At the lowest speed (1.11 m/s), V̇_E_ was different from control values only with water at the height of the carpus (*p* = 0.02). At 1.25 and 1.39 m/s, V̇_E_ values for water at the mid cannon (*p* < 0.01) and carpus levels (*p* < 0.0001) were both higher than those for the stifle at the same speed. There were no differences between the V̇_E_ values for the carpus and mid cannon levels when speeds were the same.

#### Expiratory/Inspiratory ratio

The ratio of expiratory/inspiratory durations was not significantly different between control and water values, or with changing water height or treadmill speed (Table 2). The median inspiratory period for all exercise tests was 0.92 s (0.77-1.00) and the median expiratory period was 0.75 s (0.57-0.87).

### Heart rate (HR)

Data from 41 of the 270 total time points was missing due to loss of signal. Therefore, the sample size varied from 12 to 15 horses at each time point (Table 3).

**Table 3:** Heart rate of Quarter Horses walking on a water treadmill

	Control (no water) (*n* = 15)	Mid cannon (*n* = 12)	Carpus (*n* = 15)	Stifle (*n* = 12)
1.11 m/s	55 (49–65)^a^	59 (53–73)^a^	61 (59–71)^bc^	67 (63–74)^c^
1.25 m/s	57 (50–63)^a^	62 (54–73)^b^	68 (62–76) ^bc^	67 (66–77)^c^
1.39 m/s	59 (51–64)^a^	61 (56–74)^b^	67 (63–75)^bc^	69 (65–78)^c^

Heart rate while working on a water treadmill at three water levels (mid cannon, carpus, stifle) and speeds (1.11, 1.25, 1.39 m/s) compared to control (no water – averaged over data collected on all 3 days). Median (IQR) shown in beats/min. Superscripts indicate differences between HR values at the same treadmill speed (rows). Values with the same superscript are not different from each other

At all three speeds, control (no water) HR was significantly lower than HR during exercise in water, with the exception of the lowest speed and height combination (mid cannon at 1.11 m/s). When comparing different water heights, HR was significantly greater at the height of the stifle compared to the mid cannon (*p* = 0.007) (Table 3). A correlation between HR and V̇O_2_ was not observed.

### Lactate

All blood lactate concentrations were between 0.4 and 1.7 mmol/L (0.8 mmol/L (0.7-0.9; data not shown).

## Discussion

Despite the widespread use of WTs, the physiological responses of horses working on them are not well documented and the workloads involved with this type of exercise have not been established. This study found that the presence of water, and the height of that water, had the most significant effect on workload, as measured by V̇O_2_. Furthermore, for WTs to be utilized for conditioning, high water levels must be used. However, the effect of duration of exercise was not examined, and its effect on conditioning is not known.

### Oxygen consumption (V̇O_2_) and ventilation

Both the height of the water and the belt speed influenced V̇O_2_. Oxygen consumption increased from control (no water) values up to the height of the stifle at 1.39 m/s, with one horse reaching a value of 25.1 ml/(kg.min) (Table 2). This is comparable to land treadmill data (without a slope) where V̇O_2_ values in Thoroughbreds walking at 1.60 m/s were found to be 20.2 ml/(kg.min) [17]. The comparatively small increases in V̇O_2_ were mirrored by small increases in HR (Table 3). The similarity of the relative changes in these parameters was expected, given the close association between the two that is reflected by the Fick equation. Even when exercise was undertaken with water at the level of the stifle, the increases in V̇O_2_ and HR were relatively small, indicating that this was low, submaximal intensity exercise. Whether this level of work resulted in an increase in fitness could not be assessed with this study design. Its intent was to quantitate the intensity of exercise associated with this level of commonly performed WT exercise, not to evaluate the effect of WT training on fitness. That would require evaluations under maximal conditions.

At the lowest workload conditions, horses were more erratic with their breathing, probably because the work was not sufficient to “force” them into a more rhythmic or controlled breathing strategy that reflects the level of energy expenditure [18–20]. As workload increased at the height of the stifle, horses began to breathe deeper and slower, as shown by larger V_T_ values and lower RFs (Table 2). This breathing strategy favours alveolar ventilation and gas exchange and reduces relative dead space ventilation (V_D_) when expressed in terms of V_D_/V_T_ [21]. Interestingly, RFs on the WT (~46 breaths/min) were about half way between those observed during swimming (~25 breaths/min) [22] and those of horses walking on a dry treadmill (Thoroughbreds ~ 65 breaths/min, Standardbreds ~ 79 breaths/min at 1.60 m/s) [17, 23]. The V_T_ measured in the WT was more similar to land treadmill values, with horses working in water at a median V_T_ of 4.90 L (4.53-5.68), compared to Thoroughbreds (mean V_T_ = 5.8 L) and Standardbreds (mean V_T_ = 5.0 L) [17, 23]. However, whereas V_T_ increased with increasing water level and speed, V̇_E_ peaked at the mid-cannon water level and then decreased at the stifle. The physiologic explanation for the observed differences in ventilation is not apparent from the data collected in this study. Previous work suggests that dead space ventilation during exercise can vary considerably, particularly at light or mild submaximal exercise intensities [24, 25]. Anatomic dead space in adult horses has been calculated to be ~1.3 L [25] and dead space in the facemask has been measured to be ~1.5 L. If we consider this dead space and examine alveolar ventilation (V̇_A_) instead, it would be ~ 2.8 L/breath less than the V_T_ values reported. When considered from this perspective there was no difference between V̇_A_ for work with water at the levels of the carpus and stifle, respectively, compared to the 29.6 L/min difference observed in V̇_E_. At the low exercise intensities used in this study it is unlikely that differences in V̇_E_ would affect V̇O_2_. As long as pulmonary gas exchange was optimal, which it has been shown to be at these workloads, V̇O_2_ would be determined by cardiac output and circulatory adjustments that increase the delivery of oxygen to the muscles, and the amount of oxygen being utilized by those muscles (ie, the arteriovenous oxygen content difference) and not by V̇_E_ [25, 26].

Another potential explanation for this change in breathing strategy could be due to differences in gait kinematics; however, gait analysis was outside the scope of this study. Previous studies have examined how water height changes the biomechanics of horses: With increasing water height, the percentage duration of the stance phase decreases, while the percentage duration of the swing phase increases [27]. At water heights of the carpus and ulna, stride frequency decreases and stride length increases [3]. Additional work has been conducted on back kinematics. At a water depth of the fetlock or carpal joint, the horse steps over the water, increasing the axial rotation through the back [28]. At depths of the elbow or shoulder, the axial rotation is decreased due to increased water resistance since the horse can no longer step over the water. This increases the pelvic flexion and decreases lateral bending [28]. High levels of water result in cranial thoracic extension and thoracolumbar flexion [29]. With water at the height of the carpus and ulna, the horse is forced to push through the water, resulting in an increased stride length and decreased stride frequency [2]. It is unlikely that stride frequency and respiratory rate are coupled while on a WT, as they are on land for galloping thoroughbreds. It has been established that this coupling does not occur in swimming horses [30]. Swimming horses employ a unique breathing strategy with a rapid inspiration followed by a prolonged expiration that ends in a forced push (where the expiration period is twice as long as inspiration period) [22, 31]. Under all conditions observed, inspiratory periods were longer than expiratory periods. Average duty cycle ratio (t_I_/t_tot_) of swimming horses was reported by Hobo et al. as being 0.33 [22]. In comparison, the duty cycle ratio of horses on the WT was 0.56, similar to that observed during the cool down period post-swimming and on land (0.50) [20, 22].

### Heart rate (HR)

Electrode signal was lost in 41 of the 270 measurements due to splashing (electrodes did not remain adhered to the skin), and adjustments could not be made once the horse was in the treadmill chamber. Most of the electrode losses were during the stifle water level condition (24 of the total 41 lost).

Heart rate peaked at the greatest water height and fastest speed (stifle height water level and 1.39 m/s), indicating that these conditions probably created the greatest workload. This is different from the findings of Lindner et al. who observed a plateau in HR, where the greatest HR was not observed at the greatest water height [6]. However, in that study, horses were trotted (3.5-5.5 m/s) and had much greater peak HRs (143 ± 13 bpm) than those observed in our walking horses (stifle height, 1.39 m/s: 69.0 bpm (65–78)). According to Betros et al. [32], HR_max_ of middle-aged Standardbreds (15.2 ± 0.04 years of age) is 213 ± 3 bpm. Assuming that the horses used during this study had similar HR_max_ values, working in water (1.11-1.39 m/s, mid cannon to stifle height) would produce HRs that were 27.7-32.7% of HR_max_.

Unlike high-speed land exercise, a linear relationship between HR and speed was not observed. This was probably due to the low speeds examined in this study. A linear relationship was also not observed between V̇O_2_ and HR, possibly due to increased stroke volume, redistribution of blood to muscles, and/or increased oxygen extraction by the muscles. It is important to note that the presence of water also influences HR. Depending on the water height, increasing buoyancy will in part counteract bodyweight, potentially resulting in a reduced HR. Additionally, the pressure of the water itself may increase venous return, and in turn result in a greater stroke volume [6]. Further research on cardiac responses to WT conditions is required.

### Lactate

Blood lactate concentration was measured to help assess the intensity of WT exercise. Values remained low throughout all conditions, with a median lactate concentration < 1.0 mmol/L and a peak value of 1.7 mmol/L. Similar values have been found in other WT studies [3, 7], with the exception of that reported by Lindner et al. who found values of 1.5-2.0 mmol/L using a more strenuous protocol [6]. This is well below the lactate threshold of 4.0 mmol/L (VLa4), indicating that under all conditions tested, the anaerobic contribution to energy production was not great enough to induce blood lactate accumulation. In comparison, studies conducted on swimming horses have found lactate values ranging anywhere from 1.0-10.0 mmol/L [22, 33].

## Conclusions

Currently there is a lack of equine WT conditioning protocols. For horses to achieve sufficient workloads on WTs, the present study shows that protocols at slow speeds must incorporate high water levels. Further research on the effects of WT conditioning protocols on athletic fitness of horses is necessary to provide further evidence-based guidelines about the use of WTs in horses.

## Abbreviations

ECG: Electrocardiogram
HR: Heart rate
RF: Respiratory frequency
t_E_/t_I_: Expiratory/Inspiratory ratio
V̇_A_: Alveolar ventilation
V̇_E_: Minute ventilation
V̇O_2_: Oxygen consumption
V_T_: Tidal volume
WT: Water treadmill

## Footnotes

1 Horse-Trainer Water, Horse Trainer, Bygmarken, Denmark.

2 Microsoft Office Excel 2013, Microsoft Canada, Canada.

3 Equine Field Ergometer, Department of Veterinary Clinical Sciences, Washington State Univeristy, Pullman, Washington.

4 Televet 100, Engel Engineering Service, Heusenstamm, Germany.

5 Lactate Scout+, EKF Diagnostics, Penarth, Wales.
